# AT1-receptor blockade: Protective effects of irbesartan in cardiomyocytes under hypoxic stress

**DOI:** 10.1371/journal.pone.0202297

**Published:** 2018-10-24

**Authors:** Mariarosaria Boccellino, Marina Di Domenico, Maria Donniacuo, Giuseppe Bitti, Giulia Gritti, Pasqualina Ambrosio, Lucio Quagliuolo, Barbara Rinaldi

**Affiliations:** 1 Department of Precision Medicine, Università degli Studi della Campania “Luigi Vanvitelli”, Naples, Italy; 2 Department of Biology, College of Science and Technology, Temple University, Philadelphia, PA, United States of America; 3 Department of Experimental Medicine, Section of Pharmacology, Università degli Studi della Campania “Luigi Vanvitelli”, Naples, Italy; Universita degli Studi di Roma La Sapienza, ITALY

## Abstract

Hypoxia induces myocardial injury through the activation of inflammatory and oxidative processes. The pivotal role of the renin angiotensin system (RAS) in the pathogenesis of cardiovascular diseases has been firmly established in clinical trials and practice; in fact many experimental and clinical data have highlighted that its inhibition has a cardioprotective role. Activated RAS also stimulates inflammation directly inducing proinflammatory and oxidative gene expression. This study aimed to investigate the protective role of a pre-treatment (10 and 100 μM) with irbesartan on injury induced by 24 h of hypoxia in HL-1 cardiomyocytes; in particular, we have analyzed the natriuretic peptide (BNP) expression, a biomarker able to modulate inflammatory reaction to cardiac injury and some markers involved in oxidative stress and inflammation. Our results demonstrated that a pre-treatment with 100 μM irbesartan significantly increased SOD activity and catalase expression of 15 and 25%, respectively, compared to hypoxic cells (P<0.05). On the other hand, it was able to reduce the release of peroxynitrite and iNOS protein expression of 20 and 50% respectively (P<0.05). In addition irbesartan exerts an anti-inflammatory activity reducing Toll-like receptors (TLRs)-2 and -4 mRNA expression, TNF-alpha expression and activity (20%) and increasing the expression of the cytokine IL-17 (40%) (P<0.05 vs hypoxia). Our findings also showed that BNP induced by ischemia was significantly and in a concentration-dependent manner reduced by irbesartan. The findings of our study demonstrated that the AT1 receptor antagonist irbesartan exerts a protective role in an *in vitro* hypoxic condition reducing oxidative stress and inflammation.

## 1. Introduction

Myocardial infarction (MI) is one of major cause of death and disability worldwide [1]. It occurs when coronary blood supply does not meet myocardial demand and leads to sudden necrosis of a large number of cardiomyocytes which trigger an intense inflammatory reaction. The reactive oxygen species (ROS) released during the acute phase of the ischemic damage induced detrimental effects with peculiar changes in cellular proteins and lipids, leading to cell dysfunction or death. ROS also directly induces pro-inflammatory cascades and strongly contributes to the pathogenesis of MI [2,3]. However, ROS stimulate tissue inflammation up-regulating inflammatory cytokines, e.g., tumor necrosis factor-α (TNF-α) and interleukin (IL)-6 in the ischemic region and surrounding myocardium [4]. In a previous study Baban B. et al., showed in cardiomyocytes of ischemic-reperfused hearts that the pressure overload reduced interleukin-10 but increased interleukin-17 [5]. Additionally, the excessive intracellular ROS generation may activate the Toll-like receptor (TLR) -4 signaling pathway [6]. Previous studies have shown an involvement of TLR-4 in experimental models of ischemic injury [7,8]. It is well documented that the renin-angiotensin system (RAS) is strongly involved in the acute phase of MI and contributes to its pathophysiologic sequel [9,10]. It is well known that myocardial ischemia increases angiotensin II levels. A chronic treatment with ACE inhibitors or angiotensin II receptor antagonists has been shown to reduce ischemia-reperfusion injury [11]. Irbesartan is a potent and selective antagonist of AT1 receptors localized on vascular smooth muscle cells and in the adrenal cortex and it is usually used to treat patients with mild-to-moderate hypertension and for lower blood pressure also in drug combination [12,13]. Clinical data have demonstrated in patients with high-risk of hypertension that irbesartan reduced inflammation, oxidative stress and exerted beneficial effects on metabolic syndrome [14].The inflammatory response plays an important role in patients with cardiovascular disease and may be useful in the diagnosis of apparently healthy subjects without known coronary artery disease and without conventional risk factors. Interleukin-1, -6, -17, and TNF-α are the main investigated cytokines among those which predict cardiovascular events involved in atherosclerosis [15,16]. A large number of endoplasmatic reticulum stress-associated proteins have been shown to be involved in the development of several types of cardiomyopathies. In particular, our previous study demonstrated that an altered oxido-reductive state in the diabetic heart leads to loss of cardioprotection [17]. Thus, in the present study we evaluated the anti-inflammatory and antioxidant activity of irbesartan in a murine cellular model, HL-1 cardiomyocytes, exposed to hypoxic stress. For this purpose we investigated the beneficial effects of the AT-1 receptor antagonist irbesartan on B-type natriuretic peptide (BNP), a plasmatic marker increased in patients with myocardial ischemia, on TLRs pathway and on oxidative balance.

## 2. Materials and methods

### 2.1. Cell culture

HL-1 cells, a cardiac muscle cell line derived from the AT-1 mouse atrial myocyte tumor lineage, were a gift from William C. Claycomb, and maintained according to described protocols [18]. The cells were grown in Ex-Cell 320 medium (JRH Biosciences, Lenexa, KS) with 10% heat-inactivated fetal bovine serum (BioWhittaker), 10 mg/ml insulin (Life Technologies, Grand Island, NY), 50 mg/ml endothelial cell growth supplement (Upstate Biotechnology, Lake Placid, NY), 1 mM retinoic acid (Sigma), 10 mM norepinephrine (Sigma), 100 units/ml penicillin, 100 mg/ml streptomycin (Life Technologies), and an additional 13 nonessential amino acids (Life Technologies). The cells were grown at 37°C in an atmosphere of 5% CO_2_ and 95% air at a relative humidity of approximately 95%.

### 2.2 Cell viability assay

Cell viability assay was performed after all experimental protocols. In particular, we have tested a pre- and post- treatment with three different concentrations of irbesartan (10, 50 and 100 μM, dissolved in 250 μL ethanol). 3x10^3^ cells were seeded in 96-well plates and treated with irbesartan at 37°C for 16 h, and then placed in an hypoxia chamber for 24 h. The medium was changed before bringing the cells. To perform the post-treatment, 3x10^3^ cells were exposed for 24 h to the hypoxic stress and later stimulated 16 h with 10, 50 and 100 μM irbesartan. The cells viability was measured by incubation of 3x10^3^ seeded cell/well with 3-[4,5-dimethylthiazol-2-yl]-2,5-diphenyl tetrazolium bromide (MTT) vitality stain reagent formulated in fresh culture medium. MTT solution at 10% was added to each well and incubated for about 2 h. Then, excess medium was removed and 100 μL of a solution of 1N hydrochloric acid at 10% in isopropanol was added to dissolve the formazan crystals. The mixture was shaken for about 20 min and the optical density in each well was measured using a microplate spectrophotometer (Microplate Reader Model 550, BIO-RAD, California, USA) at 570 nm. Triplicate experiments were performed for each concentration. The cell viability percentage (%) was calculated by comparison with a sample’s corresponding control.

### 2.3 Hypoxic stress

To induce an hypoxic stress condition, 3x10^3^ HL-1 cells were put in a sealed humidified chamber (Billups-Rothenburg, Del Mar, California) supplied with 5% carbon monoxide and 95% nitrogen for 24 h. Finally, a MTT assay was performed to test cell viability and the hypoxic threshold in cardiomyocytes.

### 2.4. Superoxide dismutase (SOD) assay

SOD activity was analyzed using a SOD Activity Assay Kit (BioVision, Inc., Milpitas, California) according to manufacturer’s instructions [19]. The assay is a colorimetric method to measure the rate of reduction of WST-1 reagent in a water-soluble formazan dye with superoxide anion and this rate of reduction is inhibited by SOD. Briefly 10^4^ cells were seeded in 6-well plates after treatment with fibronectin and after 24 h incubation at 37°C they were washed once and treated with irbesartan (10 and 100 μM) at 37°C for 16 h and then were put in a hypoxic chamber. Finally, SOD Kit reagents were added to cell lysates and transferred in wells of a 96-well plate and incubated for 20 min and then the absorbance was read at 450 nm. The results were used to calculate the inhibition rate and to extract the SOD activity (U/mL).

### 2.5. Griess test

10^4^ cells were seeded in 6-well plates after treatment with fibronectin and after 24 hours incubation at 37°C were washed once and stimulated with irbesartan (10 and 100 μM) at 37°C for 16 h and then were put in a hypoxic chamber. After 24 h of the hypoxic stress, the supernatants of each point were used to measure nitrite levels by the Griess reaction according to literature [20]. Briefly, 100 μL of supernatant was mixed with an equal volume of Griess reagent (0.5% sulfanilamide, 2.5% H_3_PO_4_, and 0.05% naphthylethylene diamine in H_2_O) and incubated for 10 min at room temperature. Absorbance was measured at 550 nm and compared with a standard curve using sodium nitrite.

### 2.6. Real-time PCR (RT-qPCR)

HL-1 cells were harvested and then used for RT-qPCR analysis. The total RNA was extracted from 10^4^ cells seeded in 6-well plates according to the instructions from the supplier of the Trizol reagent (Invitrogen, Milan, Italy), then quantified using NanoDrop-1000 (Thermo Scientific, USA) [21] Using cDNA obtained by reverse transcriptase of RNA extracted from cells (Bio-Rad Laboratories, Milan, Italy), the levels of BNP, TLR-2, TLR-4 and Glyceraldehyde 3-phosphate dehydrogenase (GAPDH) mRNAs were quantified by RT-qPCR using SYBR Green (Bio-Rad Laboratories, Milan, Italy). The oligonucleotide sequences are listed below; mRNA concentrations were expressed as ratio over GAPDH which was amplified as housekeeping gene.

Sense primer:

GAPDH     5’-GCATCCTGCACCACCAACTG-3’

BNP           5’-CTGAAGGTGCTGTCCCAGAT-3’

TLR-4       5’-CCCTTATTCAACCAAGAAC-3’

TLR-2       5’-CAGAGGACTCAGGAGCAGC-3’

Antisense primer:

GAPDH     3’-CACAGTCTTCTGAGTGGCAG-5’

BNP           3’-CCTTGGTCCTTCAAGAGCTG-5’

TLR-4       3’-CTGGATAAATCCAGCCACTG-5’

TLR-2       3’-GCCTTCCCTTGAGAGGCC-5’

### 2.7. Western blot analysis

10^4^ HL-1 cells were lysed by incubation on ice for 30 min with RIPA lysis buffer and 10 μL/mL leupeptin, 5 μL/mL aprotinin, 1 μmol/L pepstatin, and 10 mmol/L dithiothreitol (DTT) were added before use. After centrifugation, the supernatant was collected, and the protein concentration was measured using a commercial kit (Bio-Rad Laboratories, Milan, Italy) according to the manufacturer’s instructions. Eighty μg of protein samples were run on 10% SDS–PAGE gels and then transferred to polyvinylidene difluoride (PVDF) membranes (Bio-Rad Laboratories, Milan, Italy). Membranes were blocked for 1 h at room temperature with 5% milk in T-TBS (Tris buffer saline with 0.1% Tween 20), followed by incubation at 4°C overnight with primary antibodies against IL-17, catalase, iNOS, TNF-α, BNP (Santa Cruz Biotechnology, Milan, Italy). Membranes were then washed three times with 0.1% T-TBS solution, and incubated for 1 h at room temperature with a secondary antibody goat anti-rabbit IgG-HRP or goat anti-mouse IgG-HRP, according to the primary antibodies data sheet (Santa Cruz Biotechnology, Milan, Italy). GAPDH antibody (Santa Cruz Biotechnology, Milan, Italy) was used as an internal standard. The immunoreactive bands were visualized using an enhanced chemiluminescence system (SuperSignal West Femto Maximum Sensitivity Substrate, Pierce, Rockford, USA). The protein bands were scanned and quantitated with Gel Doc-2000 (Bio-Rad, Milan, Italy).

#### Enzyme-linked immunosorbent assay (ELISA)

TNF-alpha and IL-17 levels were measured in the supernatants from cell cultures with a commercial ELISA kit (Elabscience, Naples, Italy). The experiments were carried out according to the manufacturer’s instructions. The values are reported as pg/mg of protein. The results derived from 3 independent experiments.

### 2.8. Statistical analysis

All data are expressed as the mean with standard deviation. Student's t-test was used to determine statistical significance of the results and p-values of less than 0.05 were considered statistically significant. In addition, One-way ANOVA followed by *Dunnett’s* test was used in order to assess the variance among the groups. The alpha critical value was set as less than 0.05 to be considered significant.

## 3. Results

### 3.1. Pre-treatment with irbesartan improves viability of HL-1 hypoxic cells

**Fig 1:**
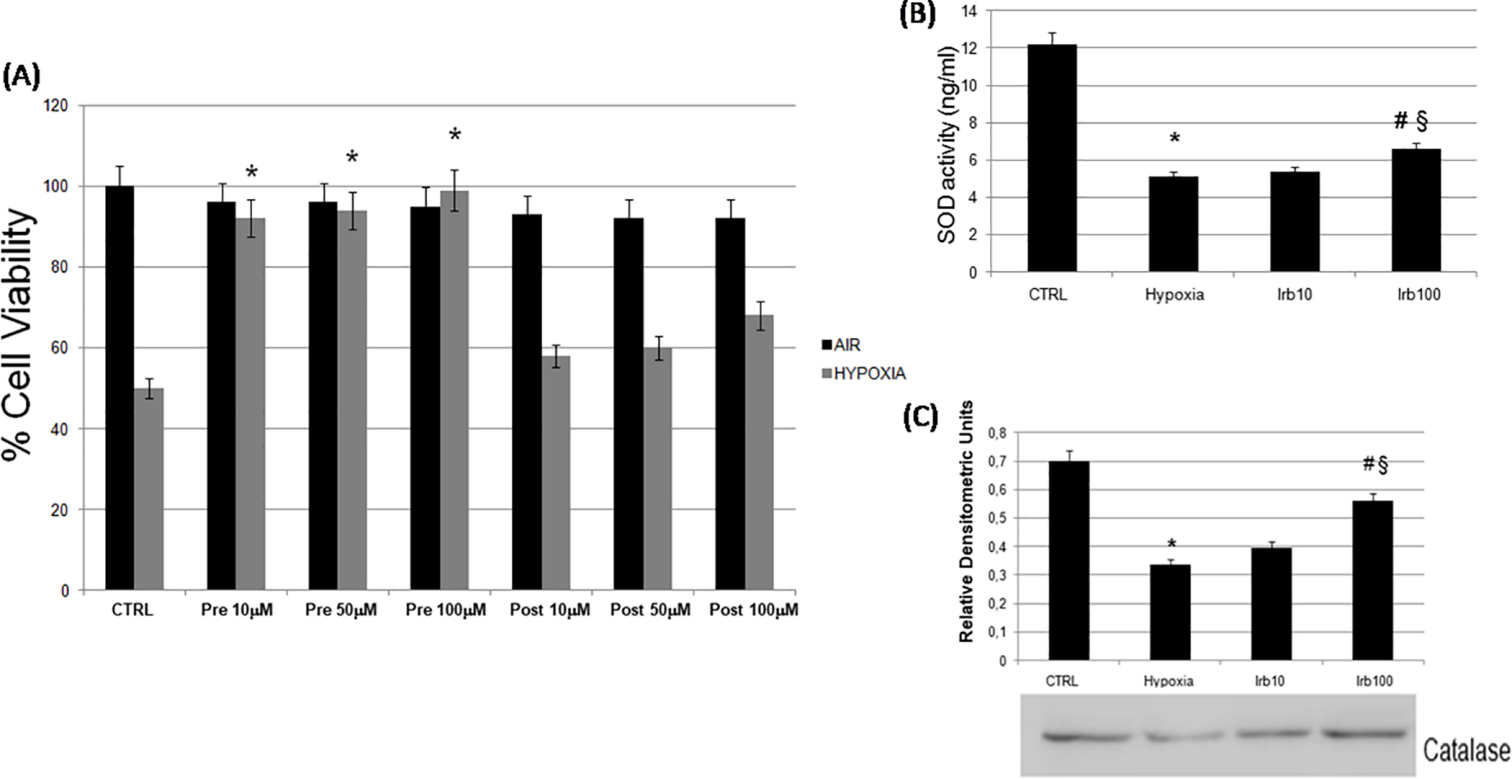
Effect of irbesartan on cell viability and on oxidative stress in HL-1 hypoxic cells. (A) Cardiomyocytes viability was determined by MTT assay in normoxia (CTRL), hypoxia alone (Hypoxia) and in combination with a pre-treatment or post-treatment with 16 h of irbesartan (Irb 10, 50, 100 μM). (B) SOD activity was determined by an ELISA assay. (C) Catalase protein expression was measured by western blot. All data are presented as mean ± S.E.M. of three independent experiments (*P<0.05 versus CTRL; #P<0.05 versus hypoxia, § P<0.05 versus irbesartan 10 μM).

We have used an *in vitro* model of simulated ischemia exposing HL-1 cardiomyocytes to hypoxic stress in a suitable chamber. The exposure of cells to hypoxia for 24 h markedly reduced cell viability to 50% compared with cells grown in standard conditions (Fig 1A). When the HL-1 cells were pre-treated with irbesartan (10, 50, 100 μM) for 16 h and then incubated in hypoxic chamber for 24 h, their viability, measured by MTT test, significantly improved by about 40% compared with untreated hypoxic cells without significant difference between the concentrations. Conversely, a post-treatment with irbesartan (10, 50, 100 μM) for 16 h causes a slight recovery of cell viability (Fig 1A). Based on these results we have chosen to stimulate the cells with irbesartan in pre-treatment, in addition, from the obtained three concentrations comparable data we decided to use only minimum and maximum irbesartan concentration (10 and 100 μM) (Fig 1A).

### 3.2. Antioxidant activity of irbesartan on hypoxic cardiomyocytes

**Fig 2:**
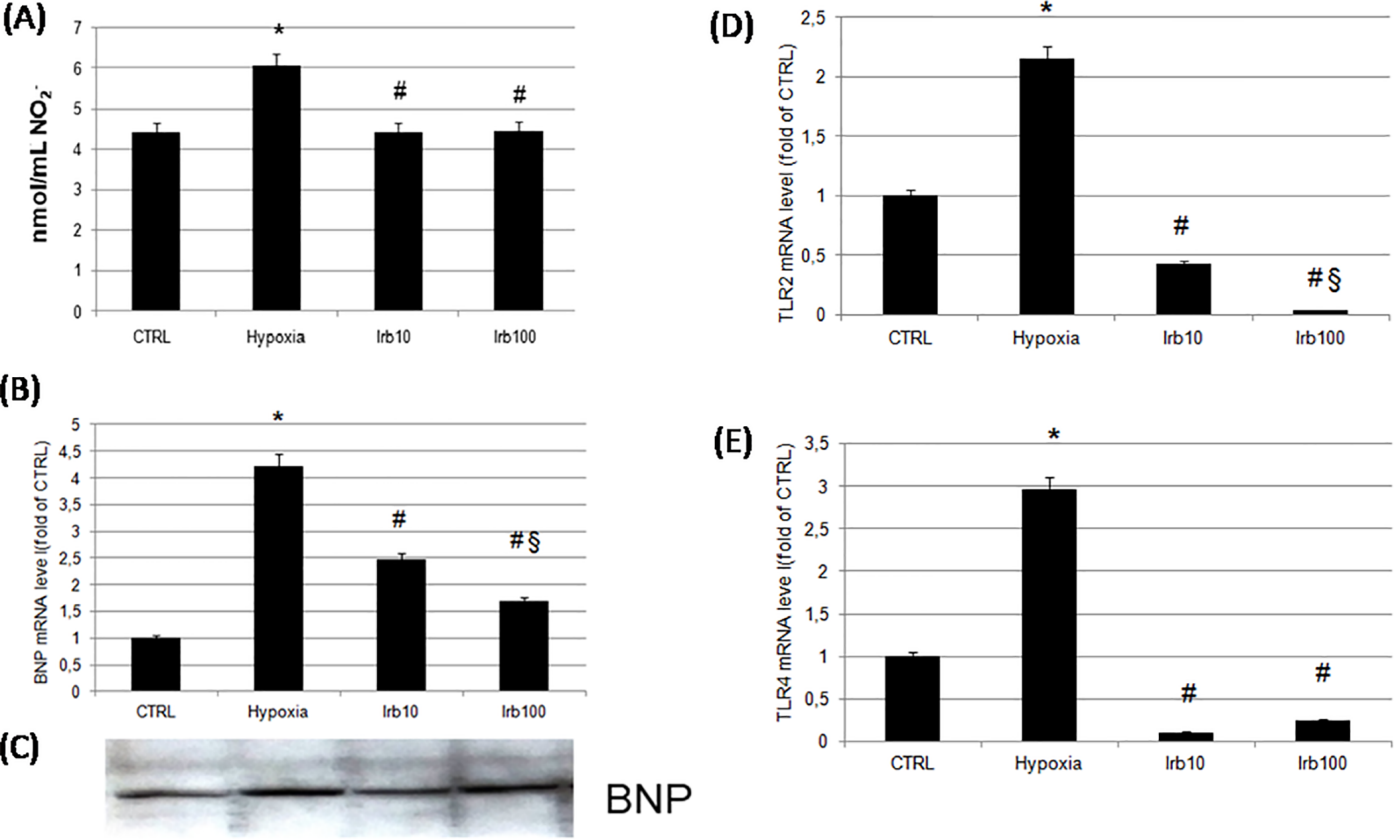
Effect of irbesartan pre-treatment on oxidative stress, on cardiac marker BNP and on TLRs mRNA expression. (A) The release of peroxynitrite was measured by Griess assay. (B) mRNA levels BNP was evaluated by RT-qPCR. (C) Western blot analysis of BNP protein, blot is representative from three independent experiments. (D) TLR2 and (E) TLR4 mRNA expressions were measured by RT-qPCR. TLR2 an TLR4 mRNA levels were normalized relative to GAPDH mRNA levels. Data represent the mean ± S.E.M. of three independent experiments (*P<0.05 versus CTRL; #P<0.05 versus hypoxia, § P<0.05 versus irbesartan 10 μM).

To investigate the role of the AT1 antagonist irbesartan on oxidative stress in hypoxic HL-1 cells we tested the effects of a pre-treatment with the two concentrations of irbesartan on SOD activity. Our data showed a significant decrease in the enzyme activity during hypoxia compared with control cells. Sixteen hours pre-treatment with irbesartan (100 μM) partially restored the enzyme activity (Fig 1B). The antioxidant effect of irbesartan was confirmed by measuring protein levels of catalase. A pre-treatment with 100 μM irbesartan significantly restore catalase expression reduced by hypoxia (Fig 1C). We also evaluated NO_2_^-^ concentrations in cardiomyocytes by using the culture medium. In hypoxic conditions as expected we observed increased NO_2_^-^ levels compared to non-stressed samples. On the other hand, a pre-treatment with irbesartan reduced significantly NO levels than in hypoxic cells (Fig 2A).

### 3.3. Effects of a pre-treatment with irbesartan on cardiomyocytes BNP expression

BNP is a diagnostic and prognostic marker in the management of patients with cardiovascular diseases and it is secreted by cardiomyocytes in response to myocardial ischemia [22]. An hypoxic condition mimics a myocardial damage induced by the lack of oxygen supply and in our experimental model 24 h of hypoxia induced in HL-1 cells a significant increase of BNP mRNA compared to control cells. A pre-treatment with 10 and 100 μM of irbesartan significantly and concentration-dependent reduced mRNA levels of this peptide (Fig 2B). To confirm this result we have performed a western blot and we have measured the protein expression of BNP active peptide. Our data showed the same profile of expression obtained by Real-time PCR (Fig 2C).

### 3.4. The AT1 receptor antagonist irbesartan reduces the inflammation induced by hypoxia negatively modulating the TLRs pathway

**Fig 3:**
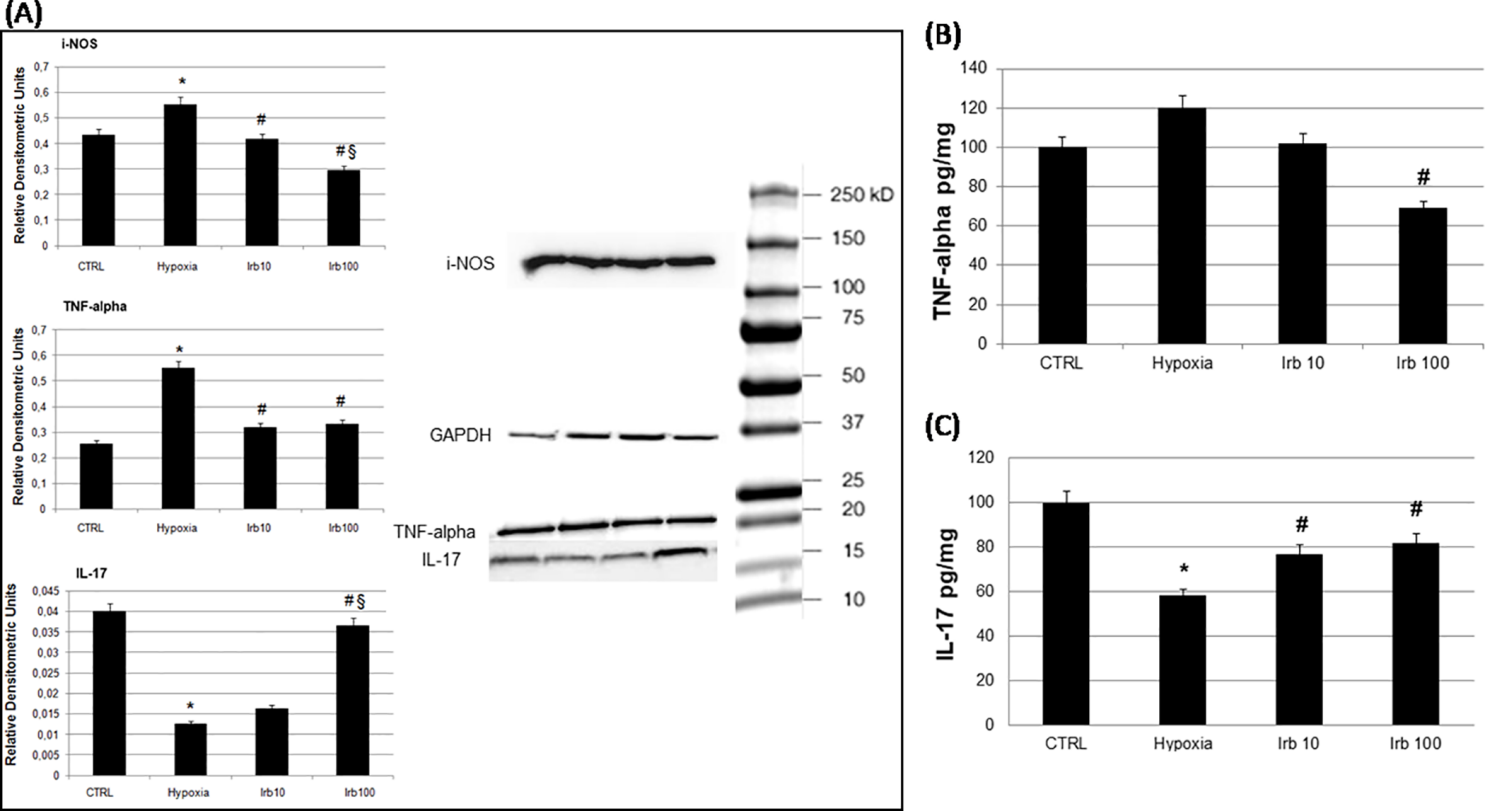
Role of irbesartan pre-treatment on inflammatory mediators and on cytokines activity. (A) iNOS TNF-alpha and IL-17 proteins expressions were measured by western blot. GAPDH was used as internal standard. Representative blot from three independent experiments. Data are expressed as relative densitometric units and represent the mean ± S.E.M. of three independent experiments (*P<0.05 versus CTRL; #P<0.05 versus hypoxia, § P<0.05 versus irbesartan 10 μM). (B) TNF-alpha and (C) IL-17 activity was measured by ELISA. Final concentration of each cytokine was expressed as pg/mg and represent the mean ± S.E.M. of three independent experiments (*P<0.05 versus CTRL; #P<0.05 versus hypoxia).

To determine the effects of irbesartan on inflammation induced by hypoxia we have chosen to measure mRNA levels of two TLRs, TLR-2 and -4, which activation by an ischemic injury aggravate tissue damage [23,24]. In particular, TLR-4 and its downstream targets such as TNF-α and i-NOS are found to facilitate the inflammatory reaction in clinical ischemic condition, i.e. myocardial infarction [25]. Our findings showed an increased mRNA of the two receptors after hypoxia. An irbesartan pre-treatment significantly reduced TLRs mRNA levels (Fig 2D and 2E). The activation of this pathway leads to an intracellular cascade of events involving inflammatory and oxidative mediators. In particular, 24 h of hypoxia induced a significant increase of inflammatory mediators such as i-NOS, TNF-α and a reduction of the antinfiammatory cytokine IL-17 (Fig 3A). An irbesartan pre-treatment (10 and 100 μM) counteracted the inflammation inducing a significant and concentration-dependent reduction of i-NOS, a reduction of TNF-α and an increase of IL-17 only at 100 μM (Fig 3A). To demonstrate and confirm that also the activity of TNF-α and IL-17 are modified by our experimental condition we performed an ELISA measurement (Fig 3B and 3C).

## 5. Discussion

Hypoxia is a major contributor to cardiac pathophysiology; a hypoxic environment induces a disproportion between oxygen supply and demand in cardiac tissue [26]. Accumulating evidences has highlighted the importance of inflammatory response and oxidative imbalance in acute myocardial infarction pathogenesis [27]. ROS overproduction induces endothelial injury and modulates the signalling of several pathways, which are known to play a crucial role in cardiovascular diseases, i.e. ischemic heart disease, stroke, kidney disease [28–30]. Previous studies have demonstrated that AT1 blockers reduced ischemic factors and the consequences of excessive ROS production; these effects were particularly investigated in experimental models of atherosclerosis and in clinical trials [31,32]. In our experimental model a pre-treatment with irbesartan, an angiotensin II receptor type AT1 antagonist, significantly reduces oxidative stress induced by hypoxia. In particular, only the highest concentration of irbesartan (100 μM) significantly improved SOD activity reduced by hypoxia; on the other hand, in a concentration-dependent manner, the pre-treatment restored the expression of another anti-oxidant enzyme, catalase. To investigate a protective role of irbesartan in cardiac hypoxia we measure BNP expression. It is well known that the inhibition of the renin-angiotensin-aldosterone system reduced plasmatic levels of BNP in cardiovascular diseases [33,34]. Plasma levels of BNP are considered one of the markers for the diagnosis of different cardiovascular diseases associated with neuroendocrine and haemodynamic changes [35]. Many clinical trials have described elevated circulating levels of BNP in patients with myocardial ischemia [36,37]. Our results showed that twenty-four hours of hypoxia markedly increased BNP levels in cardiomyocytes; a pre-treatment with irbesartan concentration-dependently restored these levels almost to the baseline. Among multiple mechanisms involved in cardiomyocyte dysfunction during ischemia, the activation of inflammatory response plays a critical role [38]. Toll-like Receptors are one of the pattern recognition receptors that initiate a defensive response to microbial invasion. Moreover, the role of TLRs in the pathophysiology of cardiovascular diseases has been widely highlighted by different authors [39]. Among these receptors TLR-2 and TLR-4 are activated following ischemic injury in cardiomyocytes [40]. The cardioprotective role of TLR4 in cardiac injury was also confirmed in TLR-4-mutated mice where the deficiency of this receptor exacerbated the cardiac dysfunction during ischemia [41]. The activation of TLR signaling through nuclear factor-κB pathway increases the expression of pro-inflammatory cytokines such as TNF-α [42,43]. For the above mentioned reasons we chose to measure the expressions of TLR- 2 and -4 after hypoxia; their expression significantly increase after 24 hours of hypoxia. A pre-treatment with irbesartan at both concentrations reported the values to baseline. To confirm the anti-inflammatory effects of irbesartan we measured the expression of two inflammatory mediators, i-NOS and TNF-α, and a specific cytokine, IL-17. As expected, hypoxia increased both inflammatory molecules but a pre-treatment with irbesartan significantly counteracted this effect. Conversely, the AT-1R blocker restored to control levels the IL-17 amount. Previous studies have shown that the biological actions of IL-17 are pro-inflammatory but there are several other studies suggesting that its role is controversial and is not yet fully understood. In fact Maione et al. show that IL-17 play a key role in sustaining chronic inflammation in autoimmune diseases. In particular, IL-17 does not initiate an inflammatory response but it is able to amplify biochemical and cellular events of this reaction [44]. Furthermore, Ke Y et al. show that IL-17 has anti-inflammatory activity and that this cytokine can suppress the development of autoimmune disease [45]. Other authors sustain that innate-derived IL-17 constitutes a major element in the altered immune response against self antigens or the perpetuation of inflammation, particularly at mucosal sites [46]. In our proposed model IL-17 could play a protective role in the presence of irbesartan in cardiomyocytes. In this perspective further studies will be needed to better understand the causative and crucial role of IL-17. Our results aimed to better understand the molecular changes of cardiomyocytes under hypoxia; any cause of hypoxia, in fact, may have a negative impact on cardiac function, and the majority of heart diseases are associated with a cardiac hypoxic stress. However, a limitation of this study is not to completely investigate the TLRs involvement; i.e. TLRs protein expression. The findings of this study add new information about the role of renin angiotensin system in the control of oxidative and inflammatory response to an experimental hypoxic condition highlighting the protective effect of the AT1 receptor antagonist irbesartan. Thus, therapeutic approaches that target components of the inflammatory and oxidative response have been investigated as potential and useful treatments for ischemic myocardial injury. Certainly a number of questions remain unsolved and further study will be needed on the hypoxia signaling to understand its pathological processes in more detail.
